# Chemotherapy-induced neuroinflammation is associated with disrupted colonic and bacterial homeostasis in female mice

**DOI:** 10.1038/s41598-019-52893-0

**Published:** 2019-11-11

**Authors:** B. R. Loman, K. R. Jordan, B. Haynes, M. T. Bailey, L. M. Pyter

**Affiliations:** 1 Center for Microbial Pathogenesis, The Research Institute at Nationwide Children’s Hospital, Columbus, Ohio USA; 2 Institute for Behavioral Medicine Research, Ohio State University, Columbus, Ohio USA; 3 Department of Psychiatry and Behavioral Health, Ohio State University, Columbus, Ohio USA; 4 Department of Pediatrics, Ohio State University, Columbus, Ohio USA; 5 Department of Neuroscience, Ohio State University, Columbus, Ohio USA

**Keywords:** Neurophysiology, Translational research

## Abstract

Chemotherapy treatment negatively affects the nervous and immune systems and alters gastrointestinal function and microbial composition. Outside of the cancer field, alterations in commensal bacteria and immune function have been implicated in behavioral deficits; however, the extent to which intestinal changes are related to chemotherapy-associated behavioral comorbidities is not yet known. Thus, this study identified concurrent changes in behavior, central and peripheral immune activation, colon histology, and bacterial community structure in mice treated with paclitaxel chemotherapy. In paclitaxel-treated mice, increased fatigue and decreased cognitive performance occurred in parallel with reduced microglia immunoreactivity, increased circulating chemokine expression (CXCL1), as well as transient increases in pro-inflammatory cytokine/chemokine (*Il-1β, Tnfα, Il-6*, and *Cxcl1*) gene expression in the brain. Furthermore, mice treated with paclitaxel had altered colonic bacterial community composition and increased crypt depth. Relative abundances of multiple bacterial taxa were associated with paclitaxel-induced increases in colon mass, spleen mass, and microglia activation. Although microbial community composition was not directly related to available brain or behavioral measures, structural differences in colonic tissue were strongly related to microglia activation in the dentate gyrus and the prefrontal cortex. These data indicate that the chemotherapeutic paclitaxel concurrently affects the gut microbiome, colonic tissue integrity, microglia activation, and fatigue in female mice, thus identifying a novel relationship between colonic tissue integrity and behavioral responses that is not often assessed in studies of the brain-gut-microbiota axis.

## Introduction

Despite recent advances in anti-cancer drugs, chemotherapeutic agents that impair cell proliferation remain the gold standard of treatment for many types of cancer. However, the combination of systemic administration of chemotherapy and its lack of cellular specificity results in numerous deleterious side effects, both behavioral (e.g., “chemobrain” cognitive impairment, fatigue)^1–3^ and gastrointestinal (GI) (e.g., diarrhea, nausea, vomiting)^4^. These side effects can be debilitating, costly, and occasionally life-threatening^5–10^, in part because they reduce adherence to cancer treatments^7,11^. Direct effects of chemotherapies on brain tissue (e.g., brain cell or myelin toxicity, oxidative stress, inflammation, neurovascular damage) are often hypothesized to underlie their effects on the brain and behavior. However, brain penetrance of various chemotherapies, including paclitaxel (a taxane chemotherapeutic)^12,13^, are limited^14,15^ suggesting that indirect mechanisms contribute to chemotherapy-induced behavioral deficits. Unfortunately, a comprehensive understanding of factors leading to these behavioral side effects are poorly understood and therefore, they remain largely untreated^16,17^.

The intestinal microbiome, a complex ecosystem of bacteria, fungi, archaea, and viruses hosted by the GI tract, may be an important mediator of these mechanisms. Specifically, the intestinal bacteriome sends biological signals (either directly or indirectly) to the brain, thereby influencing CNS homeostasis, behavior, and mood^18,19^. While changes in mood or cognitive function have not been linked to the intestinal microbiome in chemotherapy-treated cancer patients or animal models, existing studies support its plausibility. In rodents, disruptions of the gut microbiota lead to cognitive impairment and anxiety-like behavior^20–23^. Inversely, manipulating their gut microbes attenuates these behaviors^23–26^. Additionally, translational studies demonstrate that chemotherapy alters fecal bacterial communities^27,28^, which is corroborated by human clinical studies^29–33^.

Another important contributor to chemotherapy-induced behavioral side effects is the extensive, bidirectional interactions between the gut, resident microbiota, and the host immune system^34,35^. Indeed, shifts in GI microbial composition are linked to systemic inflammation^36^ and changes in neuroimmune function^37^. Relevant to this study, elevated circulating cytokines and neuroinflammation are associated with cognitive impairment, fatigue, and mood disorders in cancer patients^38,39^ and are causal in rodent cancer models^40^. Thus, here we hypothesized that the gut microbiota contributes to chemotherapy-induced neuroinflammation and behavioral changes. We examined concurrent changes in sickness, cognitive, and anxiety-like behaviors, central and peripheral immune activation, colonic histology, and bacterial community structure in mice treated with paclitaxel chemotherapy. Although paclitaxel is used to treat a wide range of cancers throughout the body^41^, it preferentially accumulates in the intestine even over tumor xenographs in mice^42^. Our data indicate that although paclitaxel leads to changes in the distal gut microbiota, which are predictive of changes in colonic histology, histological changes in colonic tissue are more predictive of behavioral and brain responses to chemotherapy than are changes in the gut microbiome.

## Results

### Chemotherapy induced sickness behaviors and increased circulating inflammatory markers

**Figure 1:**
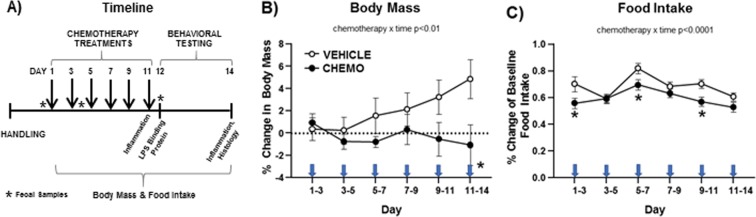
Experimental timeline and effects of chemotherapy on sickness outcomes. (**A**) Temporal sequence of chemotherapy treatments, behavioral testing, and tissue collections. (**B**) Mean ± SEM % change in body mass relative to baseline, (**C**) Mean ± SEM % change in food intake relative to baseline. Arrows indicate days of injections. n = 20/group; *p < 0.05 between chemotherapy and control groups.

**Figure 2:**
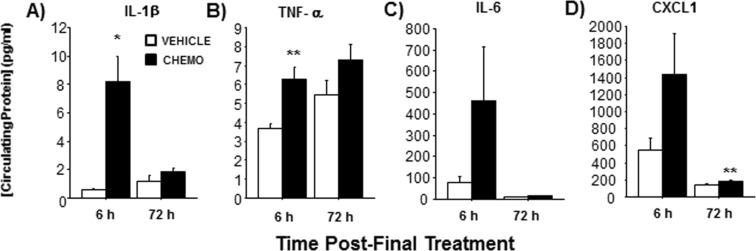
Effects of chemotherapy on circulating inflammatory markers. Mean ± SEM plasma (**A**) IL-1β, (**B**) TNFα, (**C**) IL-6, and (**D**) CXCL1 concentrations (pg/ml) 6 or 72 h post-final chemotherapy or vehicle treatment. n = 10/group (6 h); n = 20/group (72 h); *p < 0.05, **p < 0.01 between chemotherapy and control groups.

Adult, female BALB/c mice were injected according to a clinically-relevant, multicycle chemotherapy treatment (or vehicle) paradigm (Fig. 1A and see “Chemotherapy” in Methods). Chemotherapy treatment inhibited body mass growth over time (Fig. 1B; F_6, 117_ = 3.112, p < 0.01). Specifically, while vehicle-treated mice gained weight throughout the course of treatment, chemotherapy-treated mice did not. This interaction over time was driven, in part, by the significant difference between the groups on the day of the 6^th^ injection (Day 11). Chemotherapy concurrently reduced food intake (as a percentage of baseline food intake) over time (Fig. 1C; F_6,10_ = 36.5, p < 0.0001). This interaction over time was driven by measurements taken on the days of the 1^st^, 3^rd^, and 5^th^ injections (Days 1, 5, and 9, respectively). Chemotherapy treatments increased circulating IL-1β (Fig. 2A; t_5_ = 3.8, p < 0.05) and TNFα (Fig. 2B; t_18_ = 4.0, p < 0.0001) concentrations 6 h after the final injection relative to vehicle-treated controls, with a similar tendency for IL-6 and chemokine CXCL1 (Fig. 2C,D; p = 0.1 for both). This chemotherapy-induced circulating inflammation largely subsided 3 days after the final chemotherapy injection; only CXCL1 was significantly higher in chemotherapy-treated mice relative to controls at this time (Fig. 2D; t_17_ = 3.493, p < 0.005).

### Chemotherapy induced neuroinflammatory markers in a time and brain region-specific manner

**Figure 3:**
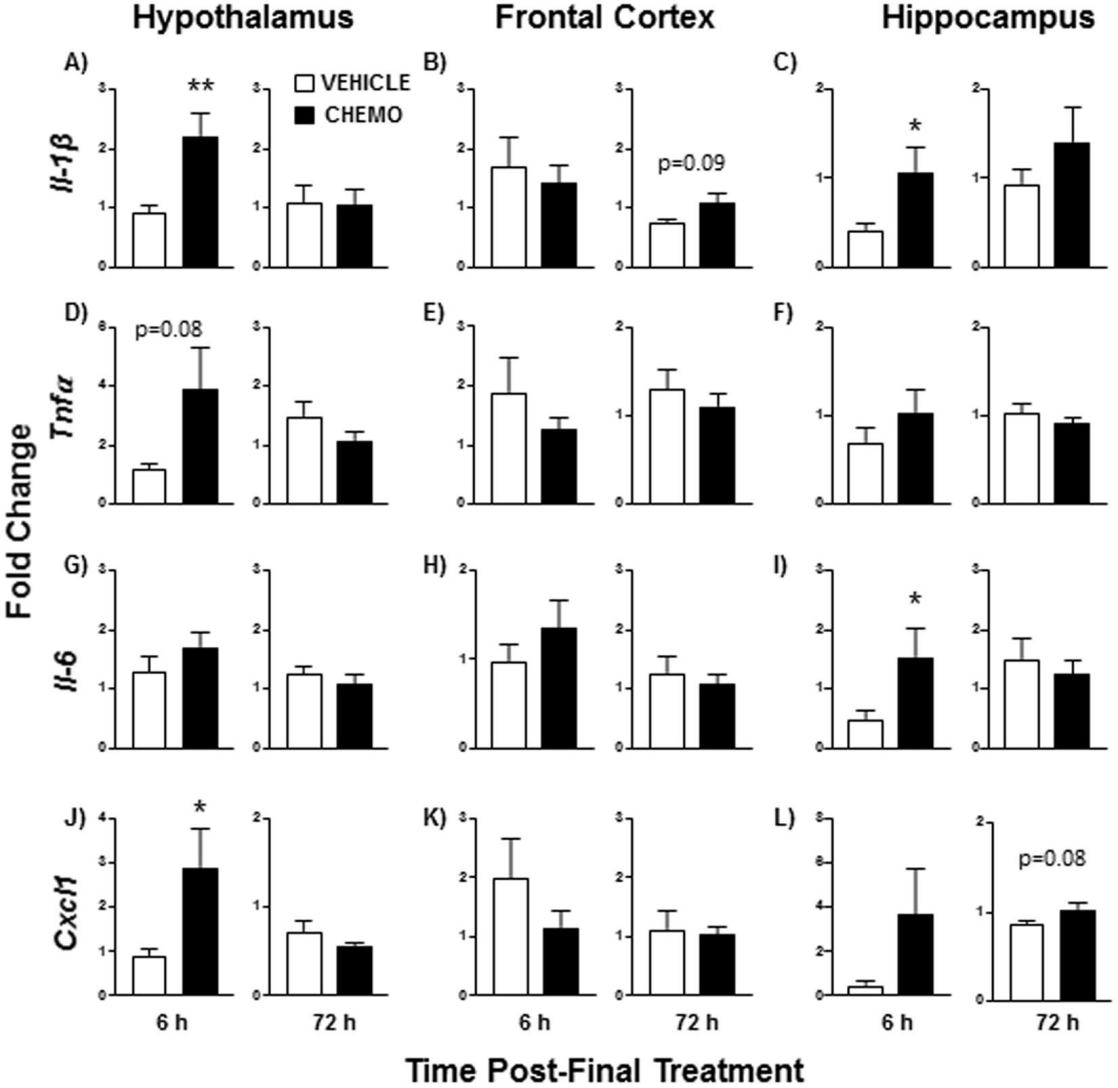
Effects of chemotherapy on brain inflammatory marker gene expression. Fold-change of (**A**–**C**) *Il-1β*, (**D**–**F**) *Tnfα*, (**G–I**) *Il-6*, and (**J**–**L**) *Cxcl1* gene expression in the hypothalamus, frontal cortex, and hippocampus 6 or 72 h following the final chemotherapy or vehicle treatment relative to vehicle controls (and normalized to *Gapdh*). n = 9–10/group; *p < 0.05, **p < 0.01 between chemotherapy and control groups.

Chemotherapy treatment increased mRNA expression of select inflammatory markers in a brain region-dependent manner. Within the hypothalamus, chemotherapy increased *Il-1β* (Fig. 3A; t_15_ = 3.2, p < 0.01) and *Cxcl1* (Fig. 3J; t_15_ = 2.2, p < 0.05) mRNA 6 h following the final chemotherapy treatment, with a similar tendency for *Tnfα* (p = 0.08). These elevations in gene expression did not persist 72 h following the final chemotherapy treatment. In the hippocampus, chemotherapy increased *Il-1β* (t_17_ = 5.9, p < 0.05) and *Il-6* (t_16_ = 4.4, p < 0.05) mRNA 6 h following the chemotherapy paradigm (Fig. 3C,F,I), with a similar tendency for *Cxcl1* (Fig. 3L; p = 0.1). Chemotherapy treatments also tended to increase hippocampal *Cxcl1* mRNA expression at 72 h post-final treatment (Fig. 3L; U = 19, p = 0.08). No significant differences were observed in the frontal cortex, although chemotherapy tended to increase gene expression of *Il-1β* (Fig. 3B; U = 28, p = 0.09) 72 h after treatment.

**Figure 4:**
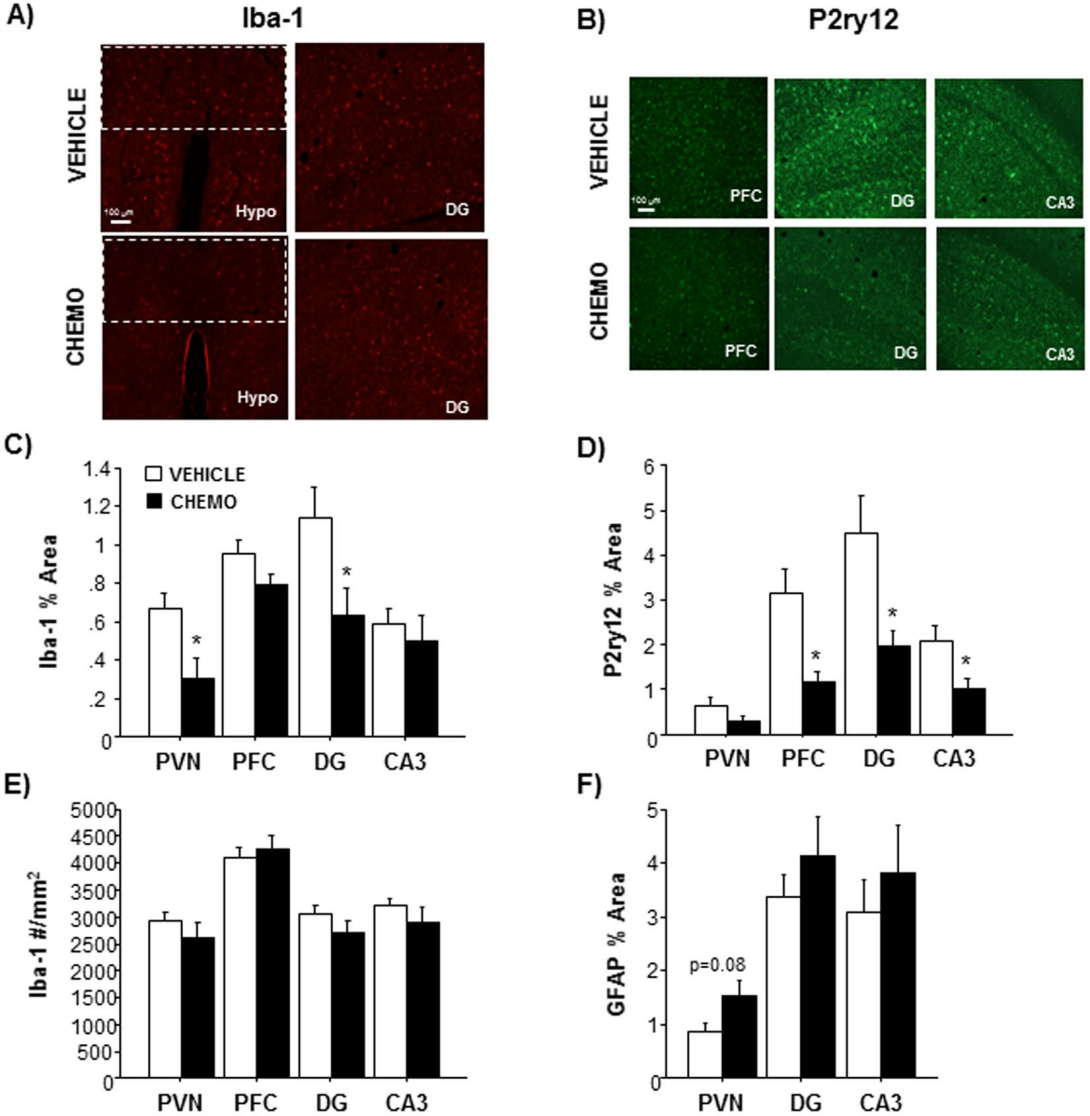
Effects of chemotherapy on microglia and astrocyte immunohistochemistry. (**A**) Representative images of Iba-1 immunohistochemistry in the PVN and DG, (**B**) Quantification of Iba-1 staining in the PVN (dotted box), PFC, DG, and CA3 regions, (**C**) Representative images of P2ry12 immunohistochemistry in the PFC, DG, and CA3, (**D**) Quantification of P2ry12 staining in the PVN, PFC, DG, and CA3 regions, (**E**) Quantification of GFAP staining in the PVN, DG, and CA3 regions. n = 8–9/group; *p < 0.05 between chemotherapy and control groups.

Iba-1 immunoreactivity, a marker of microglial activation/number, was significantly decreased in the paraventricular nucleus (PVN) of the hypothalamus (Fig. 4A,C; t_10_ = 2.771, p < 0.05) and the dentate gyrus (DG) of the hippocampus (Fig. 4A,C; t_12_ = 2.337, p < 0.05) in chemotherapy-treated mice 3 days after treatment. No differences in the number of Iba-1^+^ cells were observed in these regions (Fig. 4E, p > 0.05), indicating that the decrease in the percentage of Iba-1 immunoreactive area is not due to a decrease in microglia number. As a secondary analysis of microglial status, chemotherapy treatments reduced P2ry12 staining in the prefrontal cortex (PFC) (Fig. 4B,D; U = 11, p < 0.05), DG (Fig. 4B,D; U = 7, p < 0.05), and CA3 of the hippocampus (Fig. 4B,D; U = 12, p < 0.05) brain regions. P2ry12 staining was not significantly different in the PVN of chemotherapy treated animals (p > 0.05). GFAP staining of astrocytes also tended to increase in the PVN (Fig. 4F; t_14_ = 1.903 p = 0.08) of chemotherapy-treated animals, but no differences were observed in the DG or CA3 regions of the hippocampus.

### Chemotherapy induced fatigue and cognitive impairment

**Figure 5:**
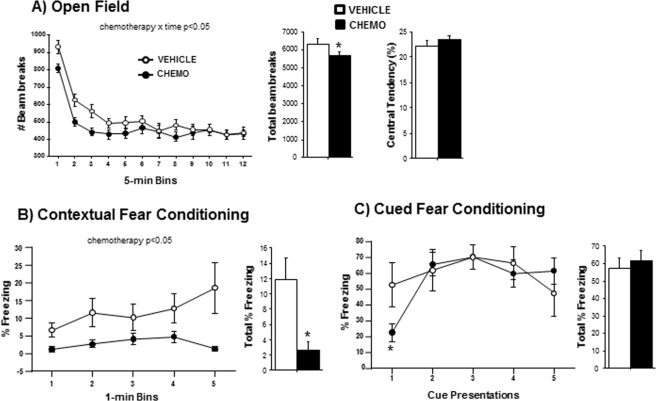
Effects of chemotherapy on fatigue, anxiety-like behavior, and memory. **(A**) Mean ± SEM central tendency and locomotion behavior in the open field 6 h following final chemotherapy or vehicle treatment, (**B**) Mean** ± **SEM percent time freezing in 1-min bins (of 5-min test) and total percent freezing in response to the conditioned contextual stimulus 2 days after final chemotherapy or vehicle treatment, (**C**) Mean ± SEM percent time freezing during each of five 30-s presentations of the conditioned cue stimulus (light) and total percent freezing 2 h after contextual testing. n = 10/group; *p < 0.05 between chemotherapy and control groups.

Chemotherapy treatments interacted with time to affect locomotion in the open field test (Fig. 5A; F_11, 198_ = 2.205, p < 0.05). While movement decreased over time in all mice, chemotherapy-treated mice reduced movement much earlier than vehicle-treated mice. No differences in anxiety-like behavior (i.e., central tendency) were observed between chemotherapy-treated and control groups (Fig. 5A; p > 0.05). In the fear conditioning assessment of contextual memory, chemotherapy consistently impaired the percent time freezing throughout the 5-min testing period (Fig. 5B; F_1,56_ = 5.9, p < 0.05) and the total percent time freezing (Fig. 5B; t_14_ = 2.4, p < 0.05). When cued conditioning was assessed, no overall differences in percent time freezing were observed between groups (Fig. 5C; p > 0.05), nevertheless, freezing in response to the very first cue presentation was reduced by chemotherapy (Fig. 5C; t_17_ = 2.1, p < 0.05). Chemotherapy did not alter the number of marbles buried in the marble burying task for obsessive-compulsive anxiety-like behavior (p > 0.05) nor the preference for light or dark zones, number of passages between zones, speed, or distance travelled in the light-dark exploration test (data not shown; p > 0.05 in all cases).

### Chemotherapy increased markers of peripheral inflammation and distal colonic crypt depth

**Figure 6:**
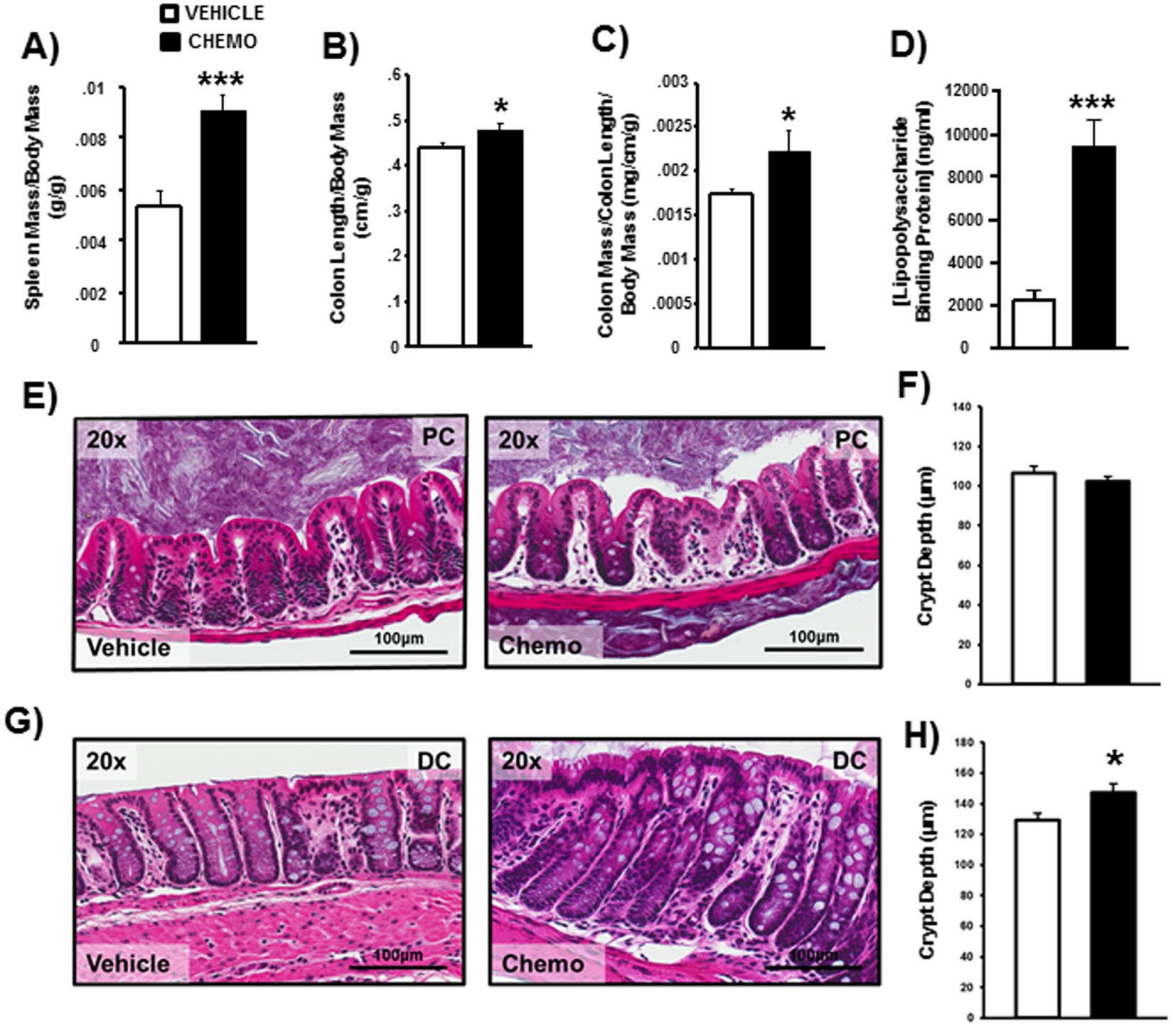
Chemotherapy altered splenic and colonic morphology. Mean ± SEM (**A**) spleen mass, (**B**) colon length, (**C**) colon mass, and (**D**) circulating lipopolysaccharide binding protein were increased by chemotherapy. While crypt depth was unaffected in the proximal colon **(E,F)**, chemotherapy increased crypt depth in the distal colon **(G,H)**. n = 8–10/group; *p < 0.05 between chemotherapy and control groups.

Chemotherapy increased spleen mass (Fig. 6A; t_36_ = 4.803, p < 0.001), colon length per body mass (Fig. 6B; t_16_ = 2.252, p < 0.05), and relative colon mass per length (Fig. 6C; t_16_ = 2.192, p < 0.05). Raw colon length was numerically higher in the chemotherapy-treated group, but did not reach statistical significance (p = 0.14). Chemotherapy also elevated circulating lipopolysaccharide binding protein (LBP) concentrations, a marker of endotoxin in the bloodstream (i.e., increased gut permeability), relative to vehicle-treated controls (Fig. 6D; t_18_ = 5.4, p < 0.0001). Morphologically, crypt depth was unchanged in the proximal colon (Fig. 6E,F), but increased by chemotherapy in the distal colon (Fig. 6G,H; t_16_ = 2.586, p < 0.05).

### Chemotherapy altered expression of limited inflammatory markers in the colon

Although expression of several classical markers of intestinal inflammation were surveyed in colonic segments, the majority (*Il-1β*, *Il-2*, *Il-6*, *Cxcl1*) were unaltered by chemotherapy (Table 1). However, expression of *Mmp9* was increased in the proximal colon (Table 1; p < 0.01), while expression of *Tnfα* was decreased in the distal colon (p < 0.05).

**Table 1:** Colonic gene expression of inflammatory markers ± SEM (arbitrary units, relative standard curve normalized to *Eef2*).

Gene	Time Post-Final Treatment	Treatment	Proximal Colon	Distal Colon
*IL-1β*	**72 h**	**VEHICLE**	1.32 ± 0.18	1.00 ± 0.07
		**CHEMOTHERAPY**	1.33 ± 0.2	1.07 ± 0.08
*Il-2*	**72 h**	**VEHICLE**	0.23 ± 0.08	0.59 ± 0.14
		**CHEMOTHERAPY**	0.17 ± 0.09	0.47 ± 0.16
*Il-6*	**72 h**	**VEHICLE**	1.32 ± 0.18	1.00 ± 0.07
		**CHEMOTHERAPY**	1.33 ± 0.2	1.07 ± 0.08
*Tnfα*	**72 h**	**VEHICLE**	1.13 ± 0.11	0.96 ± 0.07
		**CHEMOTHERAPY**	1.18 ± 0.12	**0.74 **± 0.08 *
*Cxcl1*	**72 h**	**VEHICLE**	0.59 ± 0.25	0.45 ± 0.06
		**CHEMOTHERAPY**	0.64 ± 0.28	0.51 ± 0.07
*Mmp9*	**72 h**	**VEHICLE**	0.80 ± 0.13	0.57 ± 0.04
		**CHEMOTHERAPY**	**1.39 **±** 0.13**^**#**^	0.53 ± 0.04

n = 8–10/group; *p < 0.05; ^#^p < 0.01.

### Chemotherapy decreased fecal bacterial diversity over the course of treatment

**Figure 7:**
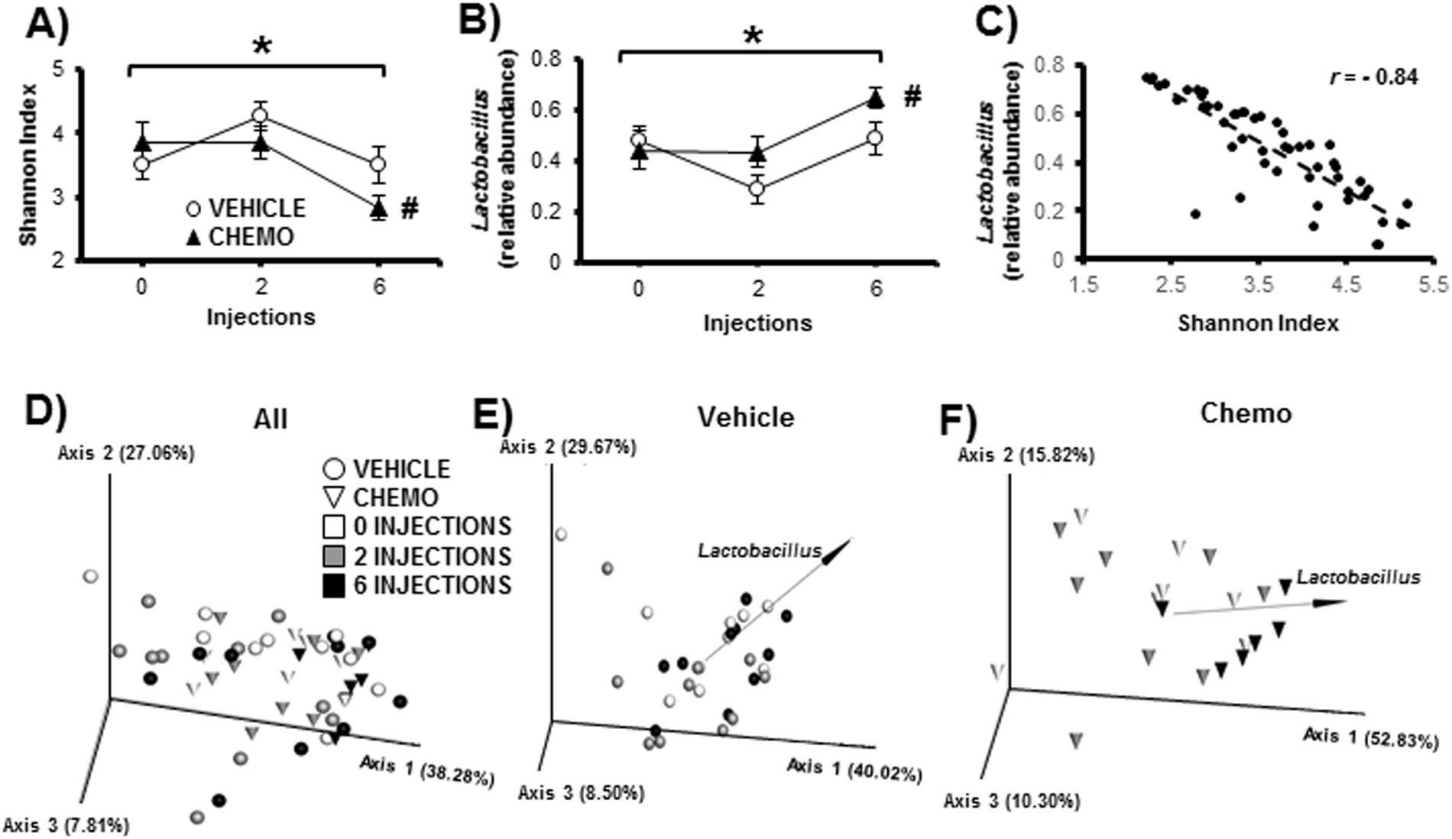
Chemotherapy altered the fecal bacteriome over time. (**A**) Alpha diversity as measured by Shannon Index decreased as an effect of time, and was decreased between baseline and post-6 injections in the chemotherapy group but not the vehicle group. (**B)** This decrease was associated with an increase in *Lactobacillus* as an effect of time and was increased between baseline and post-6 injections in the chemotherapy group but not the vehicle group and (**C)** was negatively correlated to alpha diversity. (**D)** Weighted UniFrac distances (beta diversity) differentiated between chemotherapy and vehicle groups, and (**E,F)** biplots for *Lactobacillus* (black arrows) suggest that relative abundance of this genus drive differences across time, especially in the chemotherapy group. n = 6–10/group; *p < 0.05 repeated measures ANOVA for time; ^#^p < 0.05 vs baseline.

Chemotherapy decreased fecal bacterial alpha diversity as indicated by Shannon index (a measure of taxonomic evenness; Fig. 7A; p < 0.05), and increased relative abundance of *Lactobacillus* (Fig. 7B; p < 0.05). In both cases, *post-hoc* tests indicated that values of the chemotherapy-treated animals were altered from baseline (both p < 0.05), which was not true for the vehicle-treated controls. Furthermore, Shannon index and relative abundance of *Lactobacillus* were highly inversely related (Fig. 7C; r = −0.84, p < 0.05). There were no differences in alpha diversity as indicated by total observed features (the total number of distinct sequences observed in the dataset, a measure of taxonomic richness).

Chemotherapy also altered fecal bacterial beta diversity as indicated by weighted UniFrac distances (a measure of how entire bacterial populations differ among individuals between each treatment, accounting for both taxonomic distances and relative abundances) (Fig. 7D; p < 0.05). Specifically, after the final injection of chemotherapy, bacterial populations were different compared to the baseline and post-second injection populations of both treatment groups. In the chemotherapy-treated group, points representing bacterial populations after all 6 injections visually clustered closer to the biplot for *Lactobacillus* (Fig. 7F), which was not the case for the vehicle-treated group (Fig. 7E).

### Chemotherapy altered proximal and distal colonic bacterial populations

**Figure 8:**
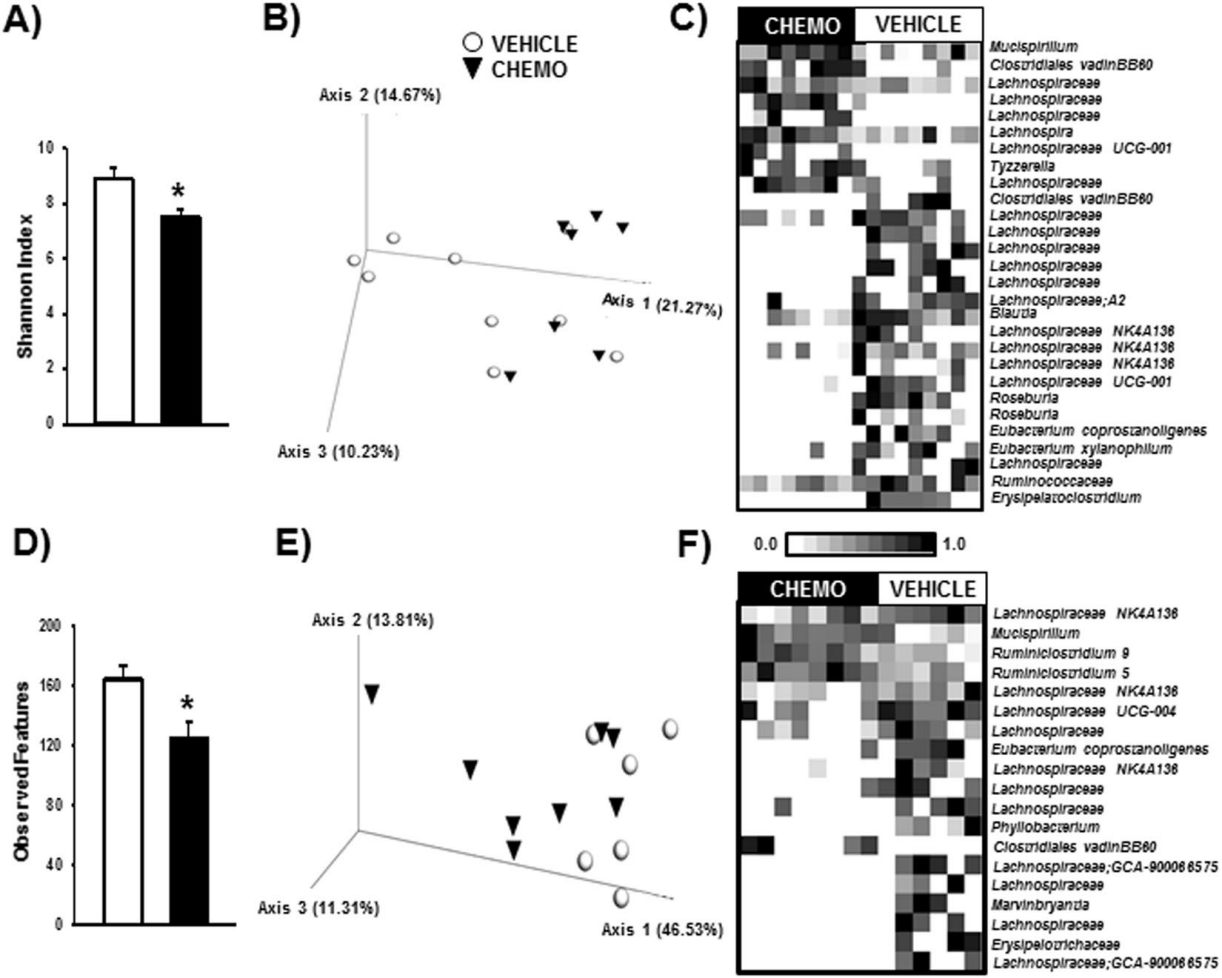
Chemotherapy altered the proximal and distal colonic bacteriomes. (**A**) Alpha diversity decreased in the proximal colon (**A**, Shannon Index) and distal colon (**D**, Total OTUs) from chemotherapy. (**B)** While unweighted UniFrac distances differentiated chemotherapy and vehicle groups in the proximal colon, (**E)** weighted UniFrac distances differentiated them in the distal colon. (**C,F)** These differences in diversity were associated with differences at the feature level. Bars represent Mean ± SEM of each treatment group. n = 6–10/group; *p < 0.05 between chemotherapy and control groups. Heatmap color intensity is normalized to 1 within each feature across subjects; p < 0.05 between chemotherapy and vehicle groups for all features presented.

In the proximal colon, chemotherapy decreased bacterial alpha diversity as indicated by Shannon index (Fig. 8A, p < 0.05), but not total observed features. Additionally, chemotherapy altered beta diversity as indicated by unweighted UniFrac distances (Fig. 8B, p < 0.05), but not weighted UniFrac distances. In the distal colon, chemotherapy decreased alpha diversity as indicated by total observed features (Fig. 8D, p < 0.05), but not Shannon index. Furthermore, chemotherapy altered beta diversity as indicated by weighted UniFrac distances (Fig. 8E, p < 0.05), but not unweighted UniFrac distances.

At the feature level, chemotherapy increased a few taxa, and decreased many taxa in both segments of the colon (Fig. 8C,F; all p < 0.05 after FDR correction). Interestingly, both segments displayed increased relative abundance of *Mucispirillum*. Chemotherapy had varying effects on the *Lachnospiraceae* family, as relative abundances of multiple features were increased and decreased. Notably, relative abundance of known butyrate-producing bacteria was decreased by chemotherapy, including *Roseburia*, *Eubacterium*, and *Erysipelotrichaceae*.

### Markers of chemotherapy in the colon and brain are related

**Figure 9:**
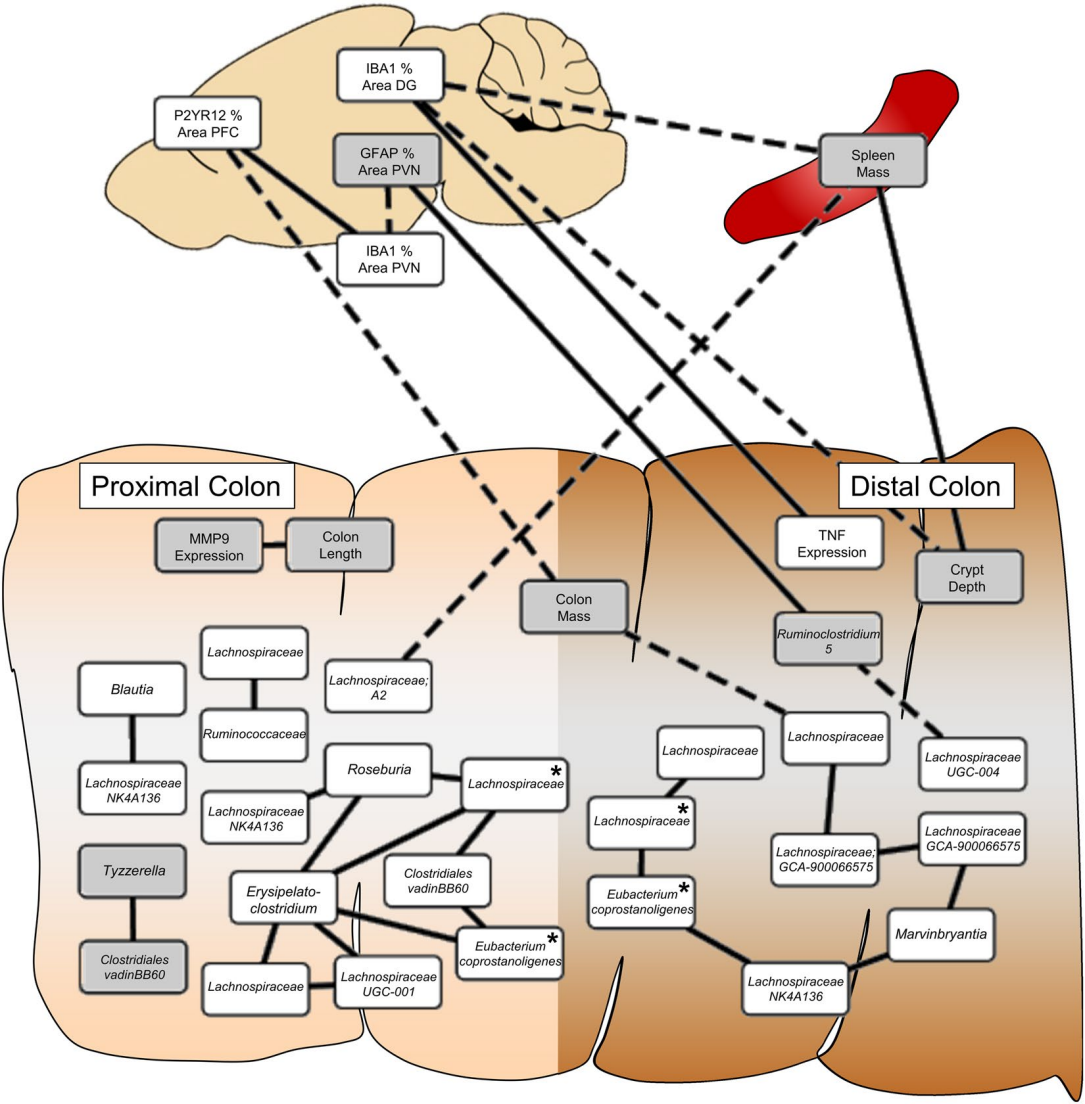
Chemotherapy-induced changes in the bacteriome, colon, and brain were related. Correlations surviving false discovery rate p-value correction are presented; magnitude of all correlation constants > 0.5. Solid lines = positive associations, dashed lines = negative associations, white boxes = higher in vehicle group, grey boxes = higher in chemotherapy group, * = identical bacterial feature between the proximal and distal colon.

All data altered by chemotherapy (bacterial relative abundance, colonic measures, spleen mass, and brain IHC) were tested for associations; the results are displayed in Fig. 9 (all p < 0.05 after FDR correction, magnitude of all r > 0.50). Relative abundances of bacterial features were highly interconnected within colon regions. Reduced *Lachnospiraceae* was related to increased colon mass, but markers of microglia activation in the brain were primarily related to changes in the colon biology (e.g., increased colon mass and crypt depth, decreased *Tnf* expression) and peripheral inflammation (spleen mass), not directly to bacterial relative abundances.

## Discussion

This study evaluated changes in behavior, inflammation, colonic morphology, and bacterial community structure induced by a multicycle paradigm of paclitaxel chemotherapy in adult female mice. Taken together, chemotherapy induced moderate sickness behaviors (anorexia, fatigue, retarded growth), impaired cognitive performance, acutely increased central and peripheral inflammation, induced evidence of endotoxin release into the circulation, altered colonic membrane morphology, as well as, reduced colonic and fecal bacterial community diversity. Importantly, this research establishes a link, for the first time, between chemotherapy-induced neurobiology and gastrointestinal and microbial changes.

In the present study, the 6-dose chemotherapy paradigm induced moderate sickness-like behavior, as indicated by reduced weight gain, food intake, and locomotion in the open field test. Mice in this age range (7–10 weeks) are still in a linear growth phase of adulthood, and thus expected to continue to increase in mass. Anorexia and weight loss are common side effects of paclitaxel^43^ and other chemotherapies in cancer patients, sometimes severe enough to switch or end treatments, and are positively associated with mortality^9,10^. Reduced locomotion may be indicative of fatigue in mice, another common side effect of chemotherapy, although further analyses to assess fatigue are necessary. Each of these sickness behaviors is associated with peripheral inflammation in chemotherapy patients^44,45^ and other rodent models^46^. Indeed, in the present study, paclitaxel-treated mice demonstrated evidence of peripheral inflammation including increased spleen mass in conjunction with increased transient concentrations of circulating LBP, IL-1β, TNFα, and CXCL1. Another study in mice failed to detect circulating inflammatory markers 2 h or 1 week post-treatment, albeit after a lower dose of paclitaxel (10 mg/kg)^43^. In paclitaxel-treated cancer patients, transient increases in circulating IL-6, IL-8, and IL-10 relative to healthy controls are likewise observed^47^. In the present study, increased and transient expression of pro-inflammatory cytokine mRNA in the brain (hypothalamus, hippocampus) 6 h after the final injection corroborated the acute response in circulation.

Congruent with our hypothesis, the observed modest chemotherapy-induced systemic inflammation was associated with neuroinflammation, albeit transient. Systemic inflammation can be transduced into the central nervous system, resulting in activation of microglia and astrocytes and increased expression of pro-inflammatory cytokines in the brain^48^. In this study, chemotherapy transiently increased expression of pro-inflammatory cytokines (*Il-1β*, *Il-6*, and *Tnfα*) and chemokine, *Cxcl1*, in the hypothalamus and/or hippocampus (6 h but not 72 h post-final injection), without affecting these markers in the frontal cortex. Within the same brain regions (e.g. periventricular nucleus, dentate gyrus, and CA3), but ~3 d later, two established immunohistochemical markers of microglial activation (Iba-1, P2ry12) decreased. Taken together, this suggests that microglia or other cytokine-producing cells in the brain (endothelial, neurons, astroglia) may be activated within 6 h of chemotherapy and induce inflammatory transcription, but after a few days result in a potentially compensatory deactivation of microglia. Of note, reduced Iba-1 staining was not associated with a decrease in microglia cells, indicating that cell death did not account for the reduced staining at this time point. Thus, this early neuroinflammatory response may be driven by oxidative stress due to chemotherapy, rather than damage-associated molecular patterns (DAMPs) from local cell death. Previous studies of this and other chemotherapies report mixed results in terms of subsequent neuroinflammation and microglial activation^49–52^. Certainly, these mixed findings may reflect the variation in time of assessment of this apparently dynamic response in the brain. Additional variation is introduced as some model paradigms include single-dose chemotherapy treatments and may also incorporate a tumor^38^. Paclitaxel-induced neuroinflammatory changes in the spinal cord and induction of neuropathic pain are more thoroughly understood. Spinal cord cytokine expression in these models is associated with increased microglial and astrocyte activation^53,54^. Overt changes in GFAP^+^ astrocytes were not observed in chemotherapy-treated mice, although more comprehensive analyses may be warranted.

Inflammation and microglial activation modulates higher order brain function, including cognitive performance, in addition to the previously discussed sickness behaviors^55^. Indeed, coincident with hippocampal inflammation, mice in this study displayed significant impairments in hippocampal-dependent contextual fear conditioning in the absence of hippocampal-independent cued conditioning. Furthermore, neuropathic pain potentially caused by this paclitaxel paradigm would have been expected to enhance this foot-shock associative learning, not impair learning, as observed. Thus, this impairment in contextual memory does not appear to be confounded by differences in allodynia, and if anything, may be underestimated. Similarly, cyclophosphamide and doxorubicin chemotherapy reduce contextual fear conditioning, although this cognitive impairment is marked by microglial activation (albeit, ~1.5 weeks later) in rats^56,57^. In other studies of cognition, different chemotherapies induce conflicting results for various cognitive functions^57,58^ although tasks in which cognition is negatively affected by chemotherapy are consistently hippocampal-dependent^57,59^ in line with that observed in the present study. Of note, the majority of these previous studies were performed in rats and only one of them used paclitaxel chemotherapy^59^.

In further support of our hypothesis, changes in colonic histology were significantly associated with the microbe-brain relationship. Given that the colon has the highest microbial density and colonization in the body, we focused on this site for our investigation of the microbe-gut-brain axis. Chemotherapy-treated mice experienced increased colonic crypt depth and expression of *Mmp9*, colon and spleen mass, and a circulating marker of intestinal barrier disruption (LBP). These changes are similar to those found in rodent models of infectious intestinal disease^60–62^. Unlike these infectious models, however, colon length was not decreased and expression of pro-inflammatory *Tnfα* and *Il-6* were not increased. In fact, colon length was increased, and *Tnfα* expression was decreased in the distal colon of paclitaxel-treated mice. Indeed, this chemotherapy-induced increase in crypt depth may be linked to changes in commensal bacteria, as the crypt hyperplasia reported in the small intestine of conventionally-raised mice treated with doxorubicin chemotherapy^63^ is absent in germ-free mice undergoing the same treatment^64^. In models of chemically-induced colitis, impairing regulatory T-cell (T_reg_) function increases colon length and mass^65^, and restoration of the T_reg_ population normalizes exacerbated increases in colonic mass^66^. Finally, the 4–5-fold increase in circulating LBP may indicate that deficits in the intestinal barrier may trigger the observed systemic (and subsequently, brain) inflammation, similar to that observed with doxorubicin chemotherapy^67^. Furthermore, in cancer patients treated with paclitaxel and platinum combination chemotherapies, gastrointestinal permeability increases relative to baseline^68^.

The effects of chemotherapy on gut microbial composition in cancer patients has not been extensively studied, but the few studies in the extant literature indicate that acute chemotherapy reduces fecal bacterial diversity or the number of bacteria able to be cultured from the stool^29–32^. In a small cohort of cancer patients receiving various chemotherapies, mean fecal *Lactobacillus* assessed via qPCR was highly variable, but numerically (not significantly) increased for 5 days following a single dose of chemotherapy^30^. This was associated with concurrent increases in circulating MMP3 and MMP9 at 2 and 5 days after treatment. In our study, 16 s DNA sequences mapped to *Lactobacillus* demonstrated high relative abundance in stool samples, which were negatively related to overall community diversity. Interestingly, we observed an increase in fecal *Lactobacillus* relative abundance immediately following the final dose of chemotherapy, and increased expression of *Mmp*9 mRNA in the proximal colon 72 h after the final chemotherapy treatment. While the relationship between *Lactobacillus* and MMPs is not yet clear, these data suggest that the colon may be an important source of increased circulating MMPs observed in humans.

In the present study, paclitaxel decreased the relative abundances of multiple bacterial taxa important to colonic health. While chemotherapy induced fluctuations in the relative abundance of members of the *Lachnospiraceae* family (i.e., relative abundances were both lower and higher based on taxa), the *Lachnospiraceae* family members that had lower representation after chemotherapy were highly inter-related with known butyrate-producing microbes. Specifically, *Roseburia*, (which is capable of producing butyrate^69^; was lower in the colon of paclitaxel-treated mice. Butyrate is a primary energy source of the colonic epithelium, and central to intestinal health^70^, and decreases in butyrate-producing bacteria are associated with behavioral comorbidities in humans and rodents^34^. While some members of *Lachnospiraceae* are capable of butyrate production^60^, some members of the *Lachnospiraceae* family are also well-recognized for their ability to initiate degradation of fiber for fermentation by other butyrate producers^71^. Thus, it is not surprising that reductions in *Lachnospiraceae* were correlated with reductions in other butyrate-producing bacteria, such as *Eubacterium* and *Erysipelotrichaceae*. The functional interactions between these bacteria and the dependence on *Lachnospiraceae* (during chemotherapy) require further investigation.

In addition to lower butyrate-producing microbes, the higher relative abundance of *Mucispirillum* is also notable for its abilities to colonize the intestinal mucus layer, scavenge reactive oxygen species, and modify host mucosal gene expression^72^. Changes in the colonic mucus layer and oxidative stress are common during colonic inflammation^73,74^, thus it is possible that chemotherapy-induced intestinal inflammation contributes to alterations in the gut microbiome through changes in mucus and oxidative stress. Although, *Mucispirillum* was not correlated with any other chemotherapy-induced bacteria, colon, or brain cell differences here.

In studies of cancer patients, the role of gut microbes in behavioral comorbidities has not yet been examined. Indeed, only one study even attempted to address associations with mood using a general quality-of-life self-reported assessment^75^. In our study, chemotherapy-induced changes in colonic bacteria were highly inter-related, but there was only one association between an individual taxa and microglia immunoreactivity in the brain. *Ruminiclostridium*, which was higher in paclitaxel-treated mice, was significantly associated with increased microglia staining in the PVN. *Ruminiclostridium* spp. are strict anaerobes that are best known for their cellulytic activities. Although their effects on host physiology have not been widely studied, our study suggests that they may have a role in the gut-brain axis. Microbial networks in the distal colon were significantly correlated with colon mass, which in turn was inversely related to microglia staining in the PFC. Similarly, crypt depth in the distal colon was inversely related to microglia staining in the DG. To date, no studies have assessed whether colonic histopathology is related to changes in the brain or behavior. However, increasing evidence suggests that increases in gut permeability contribute to anxiety and depression^76,77^. When considered with the observed increase in LBP in the blood, which is indicative of increased intestinal permeability, our study suggests that the integrity of the colonic barrier is an important mediating factor in the brain-gut-microbiota axis.

Chemotherapies have multiple, debilitating side effects, including both gastrointestinal and behavioral effects. However, because the biological underpinnings of the behavioral side effects are not well understood, they go largely untreated and result in reduced treatment compliance^7,11^. This study demonstrates that paclitaxel chemotherapy induces sickness behavior that is associated with changes in colonic tissue integrity; changes in colonic tissue integrity, in turn, are associated with alterations in the gut microbiome. These findings suggest that therapeutic strategies that target the gut microbiota, (e.g., dietary interventions, prebiotics and/or probiotics) that can attenuate the deleterious effects of chemotherapy on gut integrity^78–80^, may also be helpful for alleviating the behavioral side effects of chemotherapy.

## Materials and Methods

### Experimental design overview

Adult, female BALB/c mice were injected according to a clinically-relevant, multicycle chemotherapy treatment (or vehicle) paradigm (Fig. 1 and see “Chemotherapy” below). In one cohort of mice, fecal samples were collected thrice: the day before the first injection, and the days after the 2^nd^ and final (6^th^) injections. Behavioral testing for lethargy and affective-like behavior was conducted over the following three days; sickness behavior (anorexia, cachexia) was measured throughout the experiment. Mice were then euthanized (Day 14) and the colon was collected for microbial, histological, and gene expression analyses, the brain was collected for inflammation-related immunohistochemistry, and blood was collected for circulating cytokine/chemokine concentrations. In a second cohort, blood was sampled the day after the final injection to assess circulating lipopolysaccharide binding protein concentrations and cognitive fear conditioning was assessed during the behavioral period (Fig. 1). In a third cohort, blood and brain tissues were collected 6 h after the final injection for earlier analysis of cytokine expression.

### Animals

Female, 7–8 week old BALB/c mice (Charles River, Wilmington, MA, USA) from mixed litters were single-housed and acclimated to a 14:10 light:dark cycle (lights off at 1500 h) in a temperature-controlled vivarium (22 ± 1 °C) for 1 week. All mice were also acclimated to handling 3 times prior to experimental procedures. Standard rodent chow and water were available *ad libitum* throughout the duration of the study. All animal experiments were approved by the Ohio State University Institutional Animal Care and Use Committee and carried out in accordance with the National Institutes of Health Guide for the Care and Use of Laboratory Animals^81^. All efforts were made to minimize animal suffering and to reduce the number of mice used.

### Chemotherapy and sickness behavior

Paclitaxel (Sigma-Aldrich, St. Louis, MO, USA), a taxane microtubule-stabilizing chemotherapeutic agent commonly used to treat breast cancer, was dissolved in Cremophor-EL (1:1 EtOH) and then diluted 1:1 with sterile PBS. Each injection consisted of either 100 μl of chemotherapy (30 mg/kg; i.p.) or vehicle, representing ~80% of an i.v. dose in patients (FDA 2005) and based on a prior dose response experiment assessing weight gain and food intake (data not shown). Mice received a total of 6 doses administered every other day during the light phase, modeled after the 4–8 doses of chemotherapy typically administered every 1–3 weeks to breast cancer patients. The duration between these chemotherapy treatments was scaled down according to mouse lifespan calculations (e.g., 10 human years ≈ 2 mouse months). While it is reported to fail to cross the blood-brain barrier^12,82^, paclitaxel has been associated with cancer patient behavioral comorbidities^83–85^. Body mass and 48-h food intake was individually recorded starting two days before injections (baseline) and every injection day thereafter to assess cachexia and anorexia. Body mass and food intake were analyzed as a percent change from baseline (n = 20/group).

### Behavioral testing

All testing for anxiety-like, cognitive, and fatigue behaviors occurred during the early dark phase (1800–2100 h) under dim red safelight unless otherwise specified. When multiple tests were run, each test was separated by 24 h starting with open field, then light/dark box, then marble burying (n = 10/group).

#### Open field

Mice were placed into the center of a 16 × 16 in arena with a sparse coating of corncob bedding and allowed to explore for a period of 1 h to measure anxiety-like behavior (central tendency) and general locomotion as previously described^86^. Fatigue was operationally defined as decreased locomotion for this study. Central tendency was calculated as the percent movement in the central 4 × 4 in zone of the arena versus total arena movement. All behavior was analyzed in 5-min intervals. The apparatus was cleaned with 70% ethanol between mice.

#### Light/Dark box

A black Plexiglas box (16 × 8 in) was fitted inside half of the open field arena to create a ‘dark’ zone and overhead background lighting was used to create a ‘light’ zone on the other side. A small opening in the black box (10 × 7.7 cm, h × w) allowed for movement between the two zones. Mice were placed in the center of the light side and allowed to explore for 15 min. Total horizontal movement, time, speed, and number of passages between the two zones were tracked using the 16-photobeam system to assess both locomotion and anxiety-like behaviors.

#### Marble burying test

Similar to previously described^86^, standard mouse cages were filled with corncob bedding to a depth of 1 in. Nine marbles were cleaned with 70% EtOH and placed uniformly across the surface of the bedding. Mice were then placed in the center of the cage for 10 min. Afterwards, mice were removed and the number of marbles buried, partially buried, and unburied were recorded to assess obsessive-compulsive anxiety-like behavior^87^.

#### Fear conditioning

Automated equipment was used to assess amygdala-based memory (freezing behavior) in a test chamber with a 16 × 16 photobeam array and shock grid floor located within a larger sound-attenuating cabinet (San Diego Instruments, San Diego, CA, USA). The square test chamber (25.4 × 25.4 cm) included transparent plexiglass walls and an overhead light bulb attached to the chamber lid. Fear conditioning was run over 2 days. On day 1, mice were transported to the test chamber in a new cage and following 3 min of acclimation, mice were exposed to 5 30-s light cue pairings with a foot-shock (0.4 mA) with 1 min intervals between each light/shock pairing. Test chambers were cleaned with ethanol between each mouse. Twenty-four h later mice completed contextual, followed by cued, memory testing. For contextual testing, mice were transported in a new cage and placed in the same test chamber in which they had received the light/shock pairing the previous day. Freezing (calculated as >0.5 s without beam breaks) was measured without shock or light cue for 5 min. Two h after measuring contextual memory, mice completed cued testing for which they were transported to a novel black, octagonal chamber via a vinegar-sprayed gloved hand. Mice were acclimated to the novel chamber for 3 min and then were presented with the previously-used light cue 5 times. Freezing during each 30-s light cue presentation was measured. The chamber was sprayed with 50% vinegar to clean between mice.

### Plasma lipopolysaccharide binding protein concentrations

Blood was collected 24 h following the final chemotherapy treatment (n = 10/group). Mice were anesthetized using isoflurane and a heparin-lined capillary tube was used to collect 100–200 μl of blood from the retro-orbital vein. Whole blood was centrifuged for 20 min at 2,500 rpm at 4 °C and plasma was collected and stored at −80 °C. Plasma samples were diluted 1:500 with dilution buffer and run in duplicate in an ELISA according to the manufacturer’s instructions (Hycult Biotech, Wayne, PA, USA). All samples exceeded the minimum detection limit (0.8 ng/ml) and intra-assay variation was <10%.

### Tissue collections

All tissues were collected during the dark phase (1600–2100 h). For histological analyses, mice were euthanized with CO_2_, blood was collected transcardially, then mice were perfused with 30 ml PBS followed by 30 ml of 4% paraformaldehyde (PFA) after removing the gastrointestinal tract. Blood was collected in heparin-lined syringes and stored on ice. Whole blood was then centrifuged for 20 min at 2,500 rpm at 4 °C and plasma was collected and stored at −80 °C for cytokine analyses. Brains were removed and stored at 4 °C in 4% PFA for 24 h before being washed thrice with PBS and then transferred to a 30% sucrose solution for 24 h, or until brains sank. The brains were then frozen in isopentane and stored at −80 °C. Colon length and mass were measured prior to removing an approximately 1 cm section from both proximal and distal segments. Half of these sections were cut open lengthwise, fixed in methacarn for 24 h, and then embedded in paraffin for morphological analyses. Colonic contents were removed with sterilized tools. Colonic contents and the other half of the colon segments were snap frozen on dry ice and stored at −80 °C prior to DNA and RNA isolation (n = 8–10/group).

For brain inflammatory qRT-PCR analyses (n = 9–10/group), mice were euthanized via rapid decapitation either 6 h or 3 days after the final injection. Brains were removed and the hypothalamus was dissected and flash frozen. The rest of the brain was bisected sagittally and preserved in RNA Later at 4 °C until the hippocampus and frontal cortex were dissected (1–3 d later). All dissected brain samples were stored at −80 °C until RNA was extracted.

### Plasma cytokine/chemokine concentrations

Concentrations of circulating cytokines and chemokines (IL-1β, IL-6, TNFα, CXCL1 [KC/GRO]) were measured using customized 4-plex fluorescent immunoassays (Meso Scale Discovery, Rockville, MD, USA) chosen based on their association with chemotherapy and behavioral comorbidities and run according to the manufacturer’s instructions. Plasma samples (n = 10–20/group) were diluted two-fold, run in duplicate, and analyzed using a QuickPlex SQ instrument (Meso Scale Discovery). Values below detection limits were reported as a concentration of 0 pg/ml (IL-1β: n = 3/20 vehicle, n = 4/19 chemo; IL-6: n = 4/20 vehicle, n = 5/19 chemo). Intra-assay variation was <10% for Il-1β, IL-2, Il-6, and CXCL1 and <15% for TNFα.

### Quantitative RT-PCR

Total RNA was extracted from the hypothalamus, frontal cortex, and hippocampus (n = 9–10/group) using Qiagen RNaeasy mini kits (Qiagen, Germantown, MD, USA) and reverse transcribed as previously reported^86^. Expression of pro-inflammatory cytokines/chemokines (*Il-1β*, *Il-6*, *Tnfα*, *Il-2*, and *Cxcl1*) in the brain was assessed based on previous studies (Pyter *et al*., 2017) and the circulating cytokine results. Relative gene expression was normalized to *Gapdh* and fold-change calculated relative to respective vehicle-treated controls using the comparative CT method (2^−∆∆CT^). For colonic segments, total RNA was extracted with TRIzol^™^ reagent (Life Technologies Corporation, Carlsbad, CA, USA) as described by the manufacturer. Gene expression of *Il-1β*, *Il-6*, *Tnfα*, *Il-2*, *Cxcl1*, and matrix metalloproteinase 9 (*Mmp*9), which is well known for its role in tissue remodeling, was normalized using the relative standard curve method and normalized to eukaryotic translation elongation factor 2 (*Eef2*)^88^.

### Immunohistochemistry

Brains were sectioned at 25 μm using a cryostat and stored in cryoprotectant at −20 °C until staining. Free-floating sections were blocked with 0.5% normal donkey serum for 1 h at room temperature and stained with rabbit anti-Iba-1 (1:1000; Wako Chemicals, Richmond, VA, USA), rabbit anti-P2ry12 (1:1000; AnaSpec Inc., Fremont, CA, USA), or goat anti-GFAP (1:1000, Abcam, Cambridge, MA, USA) overnight at 4 °C. Microglial marker P2ry12 was assessed as a corroborative measure of the Iba-1 results and GFAP was used to label astroglia. Sections were washed and incubated with either Alexa Fluor 488 or Alexa Fluor 594 secondary antibodies, mounted, and coverslipped using water-soluble mounting media. Images of the prefrontal cortex, paraventricular nucleus, dentate gyrus, and CA3 of the hippocampus, brain regions that regulate cognition and sickness and anxiety-like behaviors, were acquired using an EVOS 2 microscope (Thermo Fisher Scientific, Waltham, MA, USA) and software at 20X magnification. ImageJ was used to determine percent area of staining with a threshold that covered cell bodies and processes while excluding background staining. Number of microglia/mm^2^ was determined by counting and averaging Iba-1^+^ cells from 2 sections/mouse (n = 8–9 mice/group) within each region by 3 blinded researchers.

Approximately 1 cm of colonic tissue was collected from proximal and distal halves, cut open in a proximal to distal manner, and cleared of digesta. Resulting tissue was fixed in methacarn, embedded in paraffin, and cut into 4 μm sections prior to hemotoxylin and eosin-staining on positively charged glass microscope slides. Slides were digitally imaged on the Aperio AT Turbo platform, and measured using Aperio ImageScope software (Leica Biosystems Imaging, Vista, CA, USA) by a researcher blinded to treatment groups. Crypt depth of 10 well-oriented crypts (a single line of continuous epithelial nuclei were visible from base [muscularis mucosae side] to tip [luminal side]) were measured drawing a line through the nuclei in 3–9 sections and averaged within subject for statistical comparison.

### 16S rRNA bacterial gene sequencing and analysis

Total DNA was extracted from stool and colonic contents (clinically most accessible microbiotal samples) using a QIAamp Fast DNA Stool Mini Kit with slight modifications to the manufacturer’s instructions (Qiagen, Germantown, MD, USA). Samples were incubated for 45 min at 37 °C in lysozyme buffer (22 mg/ml lysozyme, 20 mM TrisHCl, 2 mM EDTA, 1.2% Triton-x, pH 8.0), 0.1 mm zirconia beads were added, then homogenized for 150 s. Samples were incubated at 95 °C for 5 min with InhibitEX Buffer, then incubated at 70 °C for 10 min with Proteinase K and Buffer AL. Afterword, the QIAamp Fast DNA Stool Mini Kit isolation protocol was continued at the ethanol step. DNA was quantified with the Qubit 2.0 Fluorometer (Life Technologies, Carlsbad, CA) using the dsDNA Broad Range Assay Kit. Samples were diluted to 5 ng/µl and sent to the Molecular and Cellular Imaging Center (MCIC) in Wooster, OH for library preparation. Paired-end (250 nt forward and reverse) sequences of the V4-V5 16 S rRNA hypervariable region were generated on the Illumina MiSeq. Quantitative Insights into Microbial Ecology (QIIME) 2.0 was utilized for amplicon processing, quality control with DADA2, and downstream taxonomic assignment using the SILVAv132 database^89^. Sequences within samples were rarefied to balance inclusion of the most samples possible without sacrificing read depth (after visual examination of alpha rarefaction curves, samples with less than one-third of the average number of reads within that site were excluded). Fecal samples were rarefied to 2,000 reads (3 chemotherapy samples dropped), proximal colon samples were rarefied to 5,250 reads (no samples dropped), and distal colon samples were rarefied to 5,000 reads (4 vehicle samples dropped). Feature-level taxonomic differences were determined utilizing Wilcoxon tests, and metadata relationships via Spearman’s correlations in JMP v13.0.0 statistical software (SAS Institute Inc., Cary, NC, USA). Multiple hypothesis testing was FDR corrected in R (R Foundation for Statistical Computing, Vienna, Austria). The correlation network was created using Cytoscape software^90^.

### Statistical analyses

Statistical analyses of organ mass, protein concentration, gene expression, and behavior were analyzed using Student’s *t*-tests when variance was normal; otherwise, nonparametric Mann-Whitney tests were used. Repeated measures ANOVAs were used to analyze changes in body mass and food intake, as well as locomotion in the open field and freezing over time for fear conditioning. Statistical significance was reported when *p* ≤ 0.05. Statview version 5.0.1 software (Scientific Computing, Cary, NC, USA) was used for all statistics except for the sequencing analyses mentioned above.
